# Weight Fluctuation and Postmenopausal Breast Cancer in the National Health and Nutrition Examination Survey I Epidemiologic Follow-Up Study

**DOI:** 10.1155/2016/7168734

**Published:** 2016-02-03

**Authors:** Marina Komaroff

**Affiliations:** Department of Epidemiology and Biostatistics, SUNY Downstate Medical Center, Brooklyn, NY 11203, USA

## Abstract

*Objective*. The aim of this study is to investigate if weight fluctuation is an independent risk factor for postmenopausal breast cancer (PBC) among women who gained weight in adult years.* Methods*. NHANES I Epidemiologic Follow-Up Study (NHEFS) database was used in the study. Women that were cancers-free at enrollment and diagnosed for the first time with breast cancer at age 50 or greater were considered cases. Controls were chosen from the subset of cancers-free women and matched to cases by years of follow-up and status of body mass index (BMI) at 25 years of age. Weight fluctuation was measured by the root-mean-square-error (RMSE) from a simple linear regression model for each woman with their body mass index (BMI) regressed on age (started at 25 years) while women with the positive slope from this regression were defined as weight gainers. Data were analyzed using conditional logistic regression models.* Results*. A total of 158 women were included into the study. The conditional logistic regression adjusted for weight gain demonstrated positive association between weight fluctuation in adult years and postmenopausal breast cancers (odds ratio/OR = 1.67; 95% confidence interval/CI: 1.06–2.66).* Conclusions*. The data suggested that long-term weight fluctuation was significant risk factor for PBC among women who gained weight in adult years. This finding underscores the importance of maintaining lost weight and avoiding weight fluctuation.

## 1. Background

In the United States, one in eight women develops breast cancer after the age of 70 years [1]. Adult weight gain is a modifiable risk factor for postmenopausal breast cancer that can be controlled through diet modification and increased exercise [2–4]. While approximately 40% of American women attempt to lose weight [5–7], a majority of women do not maintain the lost weight [8–10]. The unsuccessful maintenance of an initial weight loss leads to weight fluctuation, which in turn promotes additional weight gain [11–15].

Each 2 kg/m^2^ increase in body mass index (BMI) is associated with 5% increased risk of postmenopausal breast cancer (Relative Risk/RR = 1.05, 95% CI: 1.03–1.07), 13% increased risk among overweight women (BMI = 25–29 kg/m^2^), and 25% among obese women (BMI ≥ 30 kg/m^2^) [3, 4]. Little is known or understood about the public health significance of weight fluctuation on the risk of postmenopausal breast cancer.

## 2. Introduction

Currently, postmenopausal breast cancer (PBC) is the most common cancer among women in the United States [1, 16–18], but the effect of weight fluctuation as risk factor for PBC has not been examined enough. Weight fluctuation also known as “yo-yo” dieting is defined as the repeated loss and regain of body weight [19]. Based on evidence from rodent and human studies, the lack of energy during initial caloric restriction induces metabolic efficiencies and fuel conservation that significantly increase tendency to weight gain [7–9, 20–22]. Dieting leads to physiological alterations that favor lipid storage, as well as abdominal accumulation of body fat. In addition, there is an increase in the rate of adipose tissue accumulation during weight regain [20–22], which is suggestive of a distinct effect on adipose tissue in the weight fluctuation process. Finally, adipose tissue is a complex and active endocrine and metabolic organ that also influences the synthesis and bioavailability of endogenous reproductive hormones [23–28]. These hormones have been shown to play a primary role in etiology and progression of PBC [23–28]. As a result, because the increase in adipose tissue is a risk factor for development of PBC [23–28], it is hypothesized that weight fluctuation increases the risk of weight gain on the development of PBC.

## 3. Materials and Methods

### 3.1. Data

Data from NHANES I Epidemiologic Follow-Up Study (NHEFS) includes subjects of 25–74 years of age who completed a medical examination at NHANES I in 1971–1975 and were followed up in 1982, 1986, 1987, and 1992. The database does not include any private information about participants and is available and open for public use. Methodology of the NHEFS has been described elsewhere [32–36].

### 3.2. Subjects

The analytic cohort was formed from women enrolled in NHANES I study. They self-reported weight at 25, 40, and 65 years of age, whereas other weight assessments were a combination of physical exams and questionnaires from the first exam in 1971–1974 through 1992 [32]. Incident cases consisted of women who were initially free of cancers at study entry but were diagnosed for the first time with the PBC during the follow-up years. Controls were selected from the subgroup of cancer-free women at study entry and during the follow-up years and matched to cases by the years of follow-up, as well as BMI status at 25 years of age defined as underweight (BMI < 18.50 kg/m^2^), normal (18.50 kg/m^2^ ≤ BMI < 24.99 kg/m^2^), overweight (25.00 kg/m^2^ ≤ BMI < 30.00 kg/m^2^), and obese (BMI ≥ 30.00 kg/m^2^) [28]. BMI was calculated using formula weight (kg)/[height (m)]^2^, where height was measured during the first physical exam in 1971–1974. Only women who had BMI assessment at age 25 and overall three or more BMI assessments before the postmenopausal breast cancer diagnosis/end of follow-up (for controls) were included into the study. Due to missing values and inconsistencies in collection of age of menopause, the breast cancer diagnosed at age 50 or greater was considered as postmenopausal based on average postmenopausal age among the US women.

### 3.3. Exclusion Criteria

Women who did not have BMI assessment at age 25 and women with less than three BMI assessments before the postmenopausal breast cancer diagnosis/end of follow-up (for control) were excluded from the study. Lastly, because the major interest in the study is to investigate the association between weight fluctuation and PBC among women who gained weight in adult years, women with lost weight were also excluded.

### 3.4. Weight Gain and Weight Fluctuation

Definition of weight fluctuation was based on the results from a simple linear regression model for each woman with her BMI regressed on age. The extent of BMI fluctuation for each participant was estimated with the root-mean-square-error (RMSE) statistic. The subset of women with a positive beta coefficient for age (*β*_age_) from a linear regression models was considered weight gainers and only weight gainers were chosen for the study. Overall, there were 79 cases and 79 controls that satisfied inclusion criteria.

### 3.5. Data of Other Risk Factors for Breast Cancer

The other postmenopausal breast cancer risk factors were included in the conditional logistic regression as the following covariates: parity or live births (0, 1, or more), age at the first child greater than 30 (yes/no), history of breast cancer in mother (yes/no), and use of hormone replacement therapy (yes/no).

In 1971–1975, weight and height were measured. In the following years, data were collected on live births, age when first child was born, history of breast cancer in first-degree female relatives (mother), and use of hormone replacement therapy.

### 3.6. Statistical Analysis

A conditional logistic regression model (PROC LOGISTIC in SAS®) was developed to assess the increase in risk of PBC for every unit increase in weight fluctuation measured by root-mean-square-error (RMSE) derived from a simple linear regression for BMI on age (from 25 years). The other risk factors for postmenopausal breast cancer, such as parity, age at the first child greater than 30, history of breast cancer in first-degree female relatives (mother), and use of hormone replacement therapy, were included in the logistic regression model as the covariates.

SAS version 9.2 was used for all analyses where significance tests were two-sided with the level of significance equaling 5%.

## 4. Results

### 4.1. Demographics and Baseline Characteristics

A total of 79 new cases of breast cancer for women of 50 years of age and older were included in this study and matched to 79 controls by the years of follow-up and BMI status at 25 years of age. The study population was weight gainers and had at least three BMI assessments before the diagnosis/end of follow-up (for controls). Distribution by 50–60, 61–70, 71–80, and >80 years of age was 51.9%, 26.58%, 17.72%, and 3.8%, respectively. The mean age at cancer diagnosis was about 63 years (standard deviation = 9.26). Most women (82.28%) had normal BMI status at age of 25 years. The proportion of women with live births “1 or more” was a little higher among controls (87.34%) versus cases (84.81%). The proportion of women with the history of breast cancer in female relatives (mother) was slightly higher among cases (83.54%) compared to controls (79.75%). More women with PBC reported that they “ever used the hormonal therapy” among cases (92.41%) versus controls (56.96%). The summary of demographic and baseline characteristics is presented in Table 1.

### 4.2. Weight Fluctuation

All 158 women self-reported weight assessments at age 25 and had three or more weight assessments before postmenopausal breast cancer diagnosis/end of follow-up (for controls). For cases, three weight assessments before cancer were collected for eight women (10.13%) while the majority of cases (88.61%) had five or more weight assessments. All controls (100%) reported five or more weight assessments for the period of follow-up. The weight fluctuation was measured as root-mean-square-error statistic from simple linear regression of dependent BMI from independent age. It was higher in cases with mean (standard deviation) that is equal to 1.51 (0.89) compared to controls 1.37 (0.86).

The assumptions underlying the regression models were checked with diagnostics statistics and plots. *R*-squares were considered acceptable. *R*-squares for controls were at the minimum of 50%. There were two cases with *R*-square less than 2%, and sensitivity analysis was conducted excluding these cases along with matched controls from the logistic regression models and did not change the results and conclusions (data is not presented).

### 4.3. Weight Gainers

It is known that weight gain is a risk factor for postmenopausal breast cancer [2–4]; however, most women who try to lose weight fail to maintain lost weight and gain weight even more [5–15]. The objective of the study was to investigate if weight fluctuation among weight gainers is an independent risk factor for PBC. Only weight gainers were chosen for this study where weight gain was defined as a positive slope (*β*_age_-coefficient > 0) from a simple linear regression of dependent variable BMI by independent age. In other words, weight gainers were women who had positive (or upward) trend in the weight change from age 25 through the adult years and to the last assessment before postmenopausal cancer diagnosis. On average, the last weight assessment was performed at about age of 60 years (standard deviation = 10.14) and was on average 2.7 (standard deviation = 2.28) years before PBC diagnosis. Due to small sample size, the investigation of pre- or postmenopausal weight gain was out of scope in this study, as well as estimation of the different levels of weight gain.

### 4.4. Weight Fluctuation and Postmenopausal Breast Cancer

The odds ratio estimate for weight fluctuation from conditional logistic regression adjusted for weight gain (*β*_age_-coefficient/slope of BMI regression on age) was 1.674 (95% confidence limit interval: 1.06–2.66) which means that each unit increase in weight fluctuation will increase the odds of getting PBC (versus odds of not getting PBC) by 67.4% among women who gain weight from age 25.

The results from conditional logistic regression adjusted for parity, first child after 30 years of age, use of hormonal therapy, history of mother's breast cancer, and weight gain (*β*_age_-coefficient/slope of BMI regression on age) made association stronger with odds ratio for weight fluctuation as 2.92 (95% CLI: 1.39–6.14). The results are presented in Table 2.

## 5. Discussion

The major finding of this study is that weight fluctuation during gaining weight in adult years is an independent risk factor for postmenopausal breast cancer.

National Task Force on the Prevention and Treatment of Obesity summarized the forty-three (43) English-language articles that evaluated the effects of weight fluctuation/cycling on humans or animals done from 1966 through 1994 [37]. The authors concluded that most studies demonstrated the association between body weight fluctuation and mortality and morbidity [37]. Nevertheless, it was hard to compare the results and drive solid conclusions because of the lack of standardized definition of weight fluctuation/cycling [37]. Search in PubMed for “weight cycling” and “cancer” revealed 21 articles with 14 related to the topic of cancer, while only three considered weight cycling as a risk factor for postmenopausal breast cancer. The conclusions of those studies are controversial. The review of those studies is summarized in Table 3.

French et al. investigated the association of weight variability and incidence of diseases including breast cancer [29]. It was the first study where weight variability was included as a separate category of weight changes during adulthood, and breast cancer in older women (55–69 years of age) was one of the outcomes under interest [29]. The authors used a sample of women from Iowa Women's Health Study (IWHS) who responded to the questionnaires and self-reported weight at ages 18, 39, 40, and 50 and current age [29]. Weight variability was defined in two ways. One way (RMSE analysis) was by the root-mean-square-error (RMSE) deviation around the linear slope of self-reported body weight at ages 18, 30, 40, and 50 [29]. And another way was by categories of the body weight changes between reported intervals of age [29]. The relative risk (RR) of breast cancer in relation to the quartiles of weight variability during adulthood (measured by RMSE) adjusted for slope of weight on age, baseline BMI, BMI^2^, age, waist/hip ratio, smoking status (never, former, and current), pack years of cigarettes, education (<high school, high school, and >high school), physical activity (low, medium, and high), alcohol (0, <4, and ≥4 g/d), marital status (yes/no), and hormone replacement (never, former, and current) did not demonstrate the association with PBC: RR (95% confidence limit interval) equals 1.05 (0.85–1.30), 0.89 (0.71–1.11), and 0.88 (0.70–1.12) for 2nd, 3rd, and 4th quartile, respectively (reference: 1st quartile of RMSE) [29]. In a second way, weight fluctuation was defined by the following categories of body weight changes derived from any two adjacent ages 18, 39, 40, and 50 and current age:* large cycle* (at least one gain ≥10% and at least one loss ≥10%) or* small cycle* (includes at least one gain ≥5% but <10% and at least one loss ≥5% but <10%) [29]. The reference category was a combined group of* stable weight* (<5% change between ages 18 and 62 y) and* small gain* (at least one gain between 5 and 10%) [29].* Large gain* category included at least one gain ≥10%; and* weight loss maintenance* group included a >10% weight loss with ±5% maintenance of reduced value.* The Others* included all other unspecified weight change patterns [29]. The relative risks for weight changes categories adjusted for age, waist/hip ratio, BMI, BMI^2^, smoking status, pack years of cigarettes, education, physical activity, alcohol, marital status, and hormone replacement demonstrated that only* large gain* was statistically significant risk for incidence of breast cancer RR (95% CI) = 1.29 (1.02–1.63) [29].* Large cycle, small cycle,* and* weight loss and maintenance* were not associated with increased risk of postmenopausal breast cancer with RR (95% CI) being 0.83 (0.60–1.14), 0.86 (0.58–1.27), and 0.81 (0.50–1.30), respectively [29].

In 2000, Trentham-Dietz et al. conducted a large case-control study of postmenopausal women to investigate the risk of breast cancer associated with different patterns of weight changes [30]. The cases were women aged 50–79 years diagnosed with invasive breast cancer in states of Massachusetts, New Hampshire, and Wisconsin (*n* = 5031) and controls (*n* = 5255) were randomly selected from driver's license files and Medicare beneficiary lists [30]. All information for height, weight, and other breast cancer risk factors was collected through the telephone interviews conducted from July 1992 through July 1995 [30]. The conditional logistic regression on age and state was used to estimate odds ratios with 95% confidence limit intervals adjusted for parity, age at first full-term pregnancy, family history of breast cancer, recent alcohol consumption, education, and age at menopause [30]. Weight cycling was defined as losing 20 or more pounds with the history of regaining at least half of it within a year [30]. The fact of weight cycling was self-reported by 18.6% among cases and 17.0% among controls [30]. Weight cyclers did not demonstrate the association with PBC relative to women who did not report any cycles (OR = 1.0, 95% CI: 0.9–1.1) [30].

In 2005, Eng et al. conducted a population-based case-control study using participants in the Long Island Breast Cancer Study Project (LIBCP: 1996-1997) to investigate the effects of patterns of body size change throughout the lifetime on postmenopausal breast cancer risk [31]. Eng et al. underscored that their study was the second that explored effect of weight fluctuation (cycling) on risk of breast cancer [31]. The authors compared weight to the control median at four time-points: 20, 30, 50, and 1 year before diagnosis (reference) where each subject was flagged “low” or “high” status below or above the control median [31]. The patterns of weight changes through life were defined as switching from “high-low” to “low-high” status or fluctuating [31]. Those groups were compared to the reference group defined as stable weight when the weight was consistently below the control median [31]. The patterns of “high-low” status consistently demonstrated the decreasing (not statistically significant) effect on risk of breast cancer, while those of “low-high” status were consistently opposite [31]. The weight cycling group demonstrated increase in risk of breast cancer with OR (95% CI) = 2.11 (1.00–4.44) [31].

The weight fluctuation/cycling were defined differently in three studies of postmenopausal women. The authors acknowledged that estimation of the risk of weight cycling is the hard task because of gaps in weight assessments in existing databases with long-term follow-up and lack of unified definition of weight cycling.

This study defines weight fluctuation similar to French et al. [29], but it is the first study with the goal of estimating the risk of weight fluctuation during adulthood among weight gainers. The rationale is that weight fluctuation provides a distinct effect on adipose tissue during weight regains and additional gains [11–15], working as an independent risk factor for PBC.

## 6. Strengths and Limitations

This is a novel study to investigate the effect of weight fluctuation during adulthood weight gain on PBC. The major strength of this study is use of national database. The major limitation is relatively small number of PBC cases. In addition, the majority of weight assessments were self-reported that introduced a recall bias (especially for 25, 40, and 65 years of age). Further studies are needed to confirm the results.

## 7. Conclusion

Understanding the relationship between weight fluctuation and postmenopausal breast cancer is very important. Many women go through weight loss programs to reduce weight gain. Yet, unsuccessful weight losses lead to weight regains and additional weight gain. This study demonstrated that weight fluctuation during weight gain is an independent risk factor for postmenopausal breast cancer. In summary, this project is one step forward to the goal of reducing the risk and the incidence of the disease. It will help to set up clear directions in weight control/management programs where successful interventions may improve health of many women.

## Figures and Tables

**Table 1:** Demographics and baseline characteristics.

Characteristics	Cases *N* = 79	Controls *N* = 79
Age of diagnosis/years of follow-up *n* (%)		
50–60	41 (51.90%)	
61–70	21 (26.58%)	
71–80	14 (17.72%)	
80 and above	3 (3.80%)	
Mean (standard deviation)	62.82 (9.26)	
Minimum-maximum	50–87	

BMI at age 25		
Mean (standard deviation)	21.11 (2.65)	
Minimum-maximum	16.83–33.77	

BMI status at age 25		
Underweight	9 (11.39%)	
Normal	65 (82.28%)	
Overweight	4 (5.06%)	
Obese	1 (1.27%)	

Live births		
0	12 (15.19%)	10 (12.66%)
1 or more	67 (84.81%)	69 (87.34%)

Age > 30 for birth of first child		
Yes (1)	4 (5.06%)	4 (5.06%)
No (2)	64 (81.01%)	68 (86.08%)
N/A	11 (13.92%)	7 (8.86%)

History of BC female relatives (mother)		
Yes	13 (16.46%)	16 (20.25%)
No	66 (83.54%)	63 (79.75%)

Have ever used hormone therapy (HRT)		
Yes	6 (7.59%)	34 (43.04%)
No	73 (92.41%)	45 (56.96%)

**Table 2:** The association between weight fluctuation and postmenopausal breast cancer.

Model	Odds ratio	95% Wald confidence limits		*p* value (Wald Chi-square)
# 1: weight fluctuation (RMSE)	1.674	1.056	2.655	0.0285^*∗*^
# 2: weight fluctuation (RMSE)	2.917	1.385	6.142	0.0048^*∗*^

Model #1 included weight gain.

Model #2 included weight gain, parity, first child at >30 years of age, history of cancer (mother), and have ever used hormonal therapy.

^*∗*^The result is significant with *α* = 0.05.

**Table 3:** Studies with weight cycling as risk factor for postmenopausal breast cancer.

Author(s)/year/[ref]	Study design population/database	Cancer Outcome	Exposure: weight cycling/analysis and covariates	Results/Conclusion	Comments
French et al., 1997 [29]	*Cohort* 1986–1992 Iowa Women's Health Study	*Postmeno* older women 55–69 years Case = 660 self- reported WT change 18–62 years	*Stable WT (ref)* (<5% change between ages 18 and 62 years) and small gain (at least one gain between 5 and 10%) *Large cycle* (at least one gain ≥10% and at least one loss ≥10%) *Small cycle* **(**at least one gain ≥5% but <10% and at least one loss ≥ 5% but <10%) *Large gain* (at least one gain ≥10%) *Weight loss maintenance* **(**a > 10% weight loss with ±5% maintenance of reduced value) *Others* (all other unspecified weight change patterns) Cox proportional hazard regression with multivariate adjustment for baseline values of age, waist/hip ratio, BMI, BMI^2^, smoking status (never, former, and current), pack years of cigarettes, education (<high school, high school, and >high school), physical activity (low, medium, and high), alcohol, marital status (yes/no), and hormone replacement (never, former, and current)	*Stable W T(ref)* *versus* *large cycle* 0.83 (0.60–1.14), *small cycle* 0.86 (0.58–1.27), *large gain* **1.29 (1.02–1.63),** *weight loss* *maintenance* 0.81 (0.50–1.30), and *others* 1.01 (0.80–1.28) *Large gainers* were at significant risk of BC *(versus stable weight)*	First study Large sample size Weight variability was defined as (1) the root-mean-square-error (RMSE) around the slope of weight on age and (2) categorical measures of weight change

Trentham-Dietz et al., 2000 [30]	*Case-Control* 50–79 years old Massachusetts, New Hampshire, Wisconsin	*Postmeno* invasive BC Case = 5031 Cont. = 5255	*Weight cycling * **:** at least 1 cycle loss of at least 20 pounds and regain back of at least half during a year Conditional logistic regression on age and state, adjusted for parity, age at first full-term pregnancy, family history of breast cancer, recent alcohol consumption, education, and age at menopause	≥1 cycle versus no cycles **OR = 1.0 (0.9–1.1)** *No association*	~18.6% cases and ~17% controls were weight cyclers

Eng et al., 2005 [31]	*Case-Control* 20–98 years old Long Island Breast CSP: 1996-1997	*Postmeno* diganosed: first primary in situ or invasive Cases = 990 Cont. = 1,006	*Weight cycling as switching from “low” to “high” *status* and back at* 4 time-points: 20, 30, 50, and 1 y before ref/diagnosis: “high” and “low” versus control medium weight (kg) Unconditional logistic regression adjusted for age at reference day (diagnosis), number of pregnancies, lactation status, nulliparity, history of breast cancer in a mother, sister, or daughter, and history of benign breast disease	**OR = 2.11 (1.00–4.44)**	Twofold increase in risk versus mainlining the “low” status
